# A Controlled Impact of Optic Nerve as a New Model of Traumatic Optic Neuropathy in Mouse

**DOI:** 10.1167/iovs.18-24773

**Published:** 2018-11

**Authors:** Ahmed S. Ibrahim, Khaled Elmasry, Ming Wan, Samer Abdulmoneim, Amber Still, Farid Khan, Abraham Khalil, Alan Saul, Md Nasrul Hoda, Mohamed Al-Shabrawey

**Affiliations:** 1 Department of Oral Biology and Diagnostic Sciences, Dental College of Georgia, Augusta University, Augusta, Georgia, United States; 2 Department of Ophthalmology and Culver Vision Discovery Institute, Medical College of Georgia (MCG), Augusta University, Augusta, Georgia, United States; 3 Department of Biochemistry, Faculty of Pharmacy, Mansoura University, Mansoura, Egypt; 4 Department of Anatomy, Faculty of Medicine, Mansoura University, Mansoura, Egypt; 5 Cellular Biology and Anatomy, MCG, Augusta University, Augusta, Georgia, United States; 6 Schepens Eye Research Institute/Massachusetts Eye and Ear & Department of Ophthalmology, Harvard Medical School, Boston, Massachusetts, United States; 7 School of Medicine, Jianghan University, Wuhan, China; 8 Department of Ophthalmology, Tulane Medical Center, New Orleans, Louisiana, United States; 9 Department of Neurology, Medical College of Georgia, Augusta University, Department of Medical Laboratory, Imaging, and Radiological Sciences, College of Allied Health Sciences, Augusta University, Augusta, Georgia, United States

**Keywords:** traumatic optic neuropathy, TON, PSTR, scotopic threshold response, controlled impact, microglia, retinal ganglion cell (RGC)

## Abstract

**Purpose:**

Traumatic optic neuropathy (TON) is the most feared visual consequence of head and ocular trauma in both military and civilian communities, for which standard treatment does not exist. Animal models are critical for the development of novel TON therapies as well as the understanding of TON pathophysiology. However, the models currently used for TON have some limitations regarding consistency and mirroring the exact pathological progression of TON in closed ocular trauma. In this study, we modified the model of controlled cortical impact and adapted it for studying TON.

**Methods:**

We defined new standardized procedures to induce TON in mice, wherein the optic nerve is reproducibly exposed to a graded controlled impact of known velocity to produce a graded deficit in retinal ganglion cell (RGC) electrophysiological functions.

**Results:**

The key results of validating this newly modified model, “controlled orbital impact (COI),” included (1) the injury parameters (velocity as well as contusion depth and time), which were quantifiable and manageable to generate a wide range of TON severities; (2) a reproducible endpoint of diminished positive scotopic threshold response (pSTR) has been achieved without the interference of surgical variability and destruction of surrounding tissues; (3) the contralateral eyes showed no significant difference to the eyes of naïve mice, allowing them to be used as an internal control to minimize interindividual variability among mice; and (4) the occurrence of injury-associated mortality and/or ocular comorbidity was rare.

**Conclusions:**

Taken together, this model overcomes some limitations of prior TON mouse models and provides an innovative platform to identify therapeutic targets for neuroprotection and/or neurorestoration following traumatic ocular injury.

Traumatic optic neuropathy (TON) is the most feared visual consequence of head and ocular trauma in both military and civilian communities, for which standard treatment does not exist.^1^ Clinically, patients with TON present with a variable degree of visual deficits ranging from decreased visual acuity to total loss of light perception, which may occur unilaterally or bilaterally.^2^ Its incidence ranges from 0.5% in all closed (nonpenetrating) head injuries^3^ to 2.5% in all maxillofacial trauma.^4^ Motor vehicle and bicycle accidents account for the majority of cases, followed by falls and assaults.^5^

In TON pathophysiology, the optic nerve can be injured either directly or indirectly.^6^ Direct optic nerve injury usually occurs from the disruption of the optic nerve per se by penetrating orbital trauma, bone fragments, or hematomas inside or outside the optic canal. This is in contrast to indirect TON, which results from the transmission of forces and/or trophic factors from closed trauma to the optic nerve without any evident injury to the adjacent tissue structures.^7^ Indirect injuries to the optic nerve are the most common causes of TON and are associated with rapid deceleration events, recreation collisions, or blows from hitting the head against a solid object.^8^ As such, children, soldiers, and victims of car crashes are the individuals most at risk for indirect TON.

Treatment of indirect TON has long been a subject of debate. The therapeutic approaches have included high-dose corticosteroids and/or decompression surgery^9^; however, recent studies have documented no apparent benefits.^1,7,10^ These therapeutic limitations highlight the need for novel pharmacologic interventions.

Animal models are critical for the development of novel TON therapies as well as the understanding of TON pathophysiology. The development of such experimental models requires evaluating the response of retinal neurons and nonneuronal glial cells to trauma. Retinal ganglion cells (RGC) are the most susceptible neurons to optic nerve damage because their axons form the optic nerve and are the primary output neurons of the retina that transmit visual signals to the brain.^11^ Therefore, assessing RGC survival and function is a central part for developing relevant animal models of TON. The expression level of the transcription factor Brn3a, which is expressed by the vast majority of RGCs,^12^ becomes a commonly used measure to assess RGC survival in various models of retinal injury,^13–15^ while measuring the amplitude of positive scotopic threshold response (pSTR), which has major ganglion cell contributions, becomes a frequently used electroretinography (ERG) parameter to measure the functionality of RGCs.^16^ The progression of RGC loss is also generally associated with the activation of retinal glial cells (Müller cells, astrocytes, and microglia), which plays an active decisive role for eventual retina adaptation or degeneration in response to trauma or injuries.^17^ Glial fibrillary acidic protein (GFAP) and ionized calcium binding adaptor molecule (Iba)-1 are routinely used markers for the reactivity of Müller/astrocytes and microglia, respectively.^17^

There are four currently used models for TON, optic nerve crush (ONC),^18^ optic nerve axotomy,^19^ blast injury,^20^ and the very recent sonication-induced TON (SI-TON)^21^; however, each reported model has some limitations regarding consistency and mirroring the exact pathological progression of indirect TON. The ONC model causes damage of adjacent tissues and deals with direct TON wherein the injuring trauma cannot be directly quantified at the site of the lesion, but is rather semiquantitatively referred to as distance between branches of a forceps.^22^ The use of forceps to induce the trauma introduces a source of variation, and consequently self-clamping forceps are often used to address this potential variable. The intrinsic spring action within the forceps is understood to be a source of a constant and consistent impact on the optic nerve. The exact duration of the impact, however, is a potential source of variability. In optic nerve axotomy, the protocol involves a complete transection of the optic nerve. Although this is useful for examining the consequent effects of this type of damage to other parts of the nervous system, it does not provide a very useful model for examining preventative therapies or treatments for indirect optic nerve trauma. Furthermore, in the axotomy model, there is no opportunity for rescue or attenuation of the inflammatory response.^19^ In the ocular blast model, high mortality rates (∼25%–50%) have been reported.^20^ In the SI-TON model, wherein a microtip probe of the sonifier is placed on the supraorbital ridge to deliver ultrasonic pulses, the difference in the sound speed between air and bone makes the focusing of sound waves in such a narrow space more unpredictable.^21^

In order to address some of these aforementioned issues, a novel controlled method for inducing indirect TON in rodents is proposed. In this method, we defined standardized procedures to induce indirect TON in mice using a controlled-impactor model. This model has the advantages of simplicity, precision control, and flexibility to generate reliable and reproducible results. The severity of injury, as assessed by pSTR of the ERG, which is thought to be a measure of ganglion cell response, is determined depending on the velocity of the impactor. The association of the velocity of the impactor (2 vs. 3 m/s) with ganglion cell function outcome should help to design experiments that address specific scientific questions, such as evaluation of severe versus mild TON, how a therapeutic intervention successfully improves TON, and how specific genetic manipulations affect the TON outcome.

## Materials and Methods

### Animal Preparation

Eight- to ten-week-old C57BL/6J wild-type mice were purchased from JAX Mice & Services (Bar Harbor, ME, USA) and maintained in an American Association for Accreditation of Laboratory Animal Care (AAALAC)-approved animal services vivarium at Augusta University. All procedures were approved by the Institutional Animal Care and Use Committee in accordance with ARVO Statement for the Use of Animals in Ophthalmic and Vision Research. Mice were fed ad libitum and housed in a temperature and humidity-controlled room under 12:12-hour light-dark cycle.

### Impact System Device

**Figure 1:**
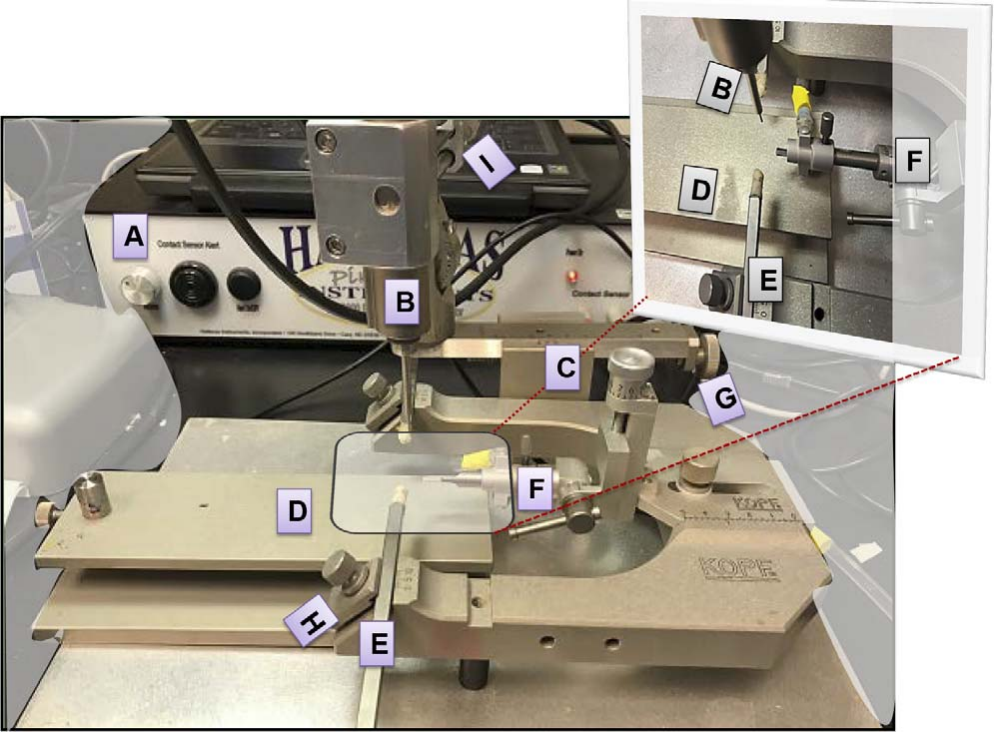
Image of the controlled impact ocular injury device. (A) The control box (PinPoint 3000 instrument) is connected to a laptop to set the impact parameters, including velocity, contusion depth, and contusion time; (B) the automated impactor; (C) articulated supported arms; (D) stereotactic frame; (E) ear bars; (F) bite plate; and (G–I) micromanipulators.

A commercially available controlled impact device (PinPoint PCI3000 Precision Cortical Impactor; Hatteras Instruments, Cary, NC, USA) integrated with PCI3000 software was used with some modifications to adapt it to TON needs. The impact system comprises a control box (Fig. 1A) to set the impact parameters, including velocity, contusion depth, and contusion time, an automated impactor (Fig. 1B), articulated supported arms (Fig. 1C), stereotactic frame (Fig. 1D), ear bars (Fig. 1E), bite plate (Fig. 1F), and micromanipulators (Figs. 1G–I).

### Experimental Procedure

**Figure 2:**
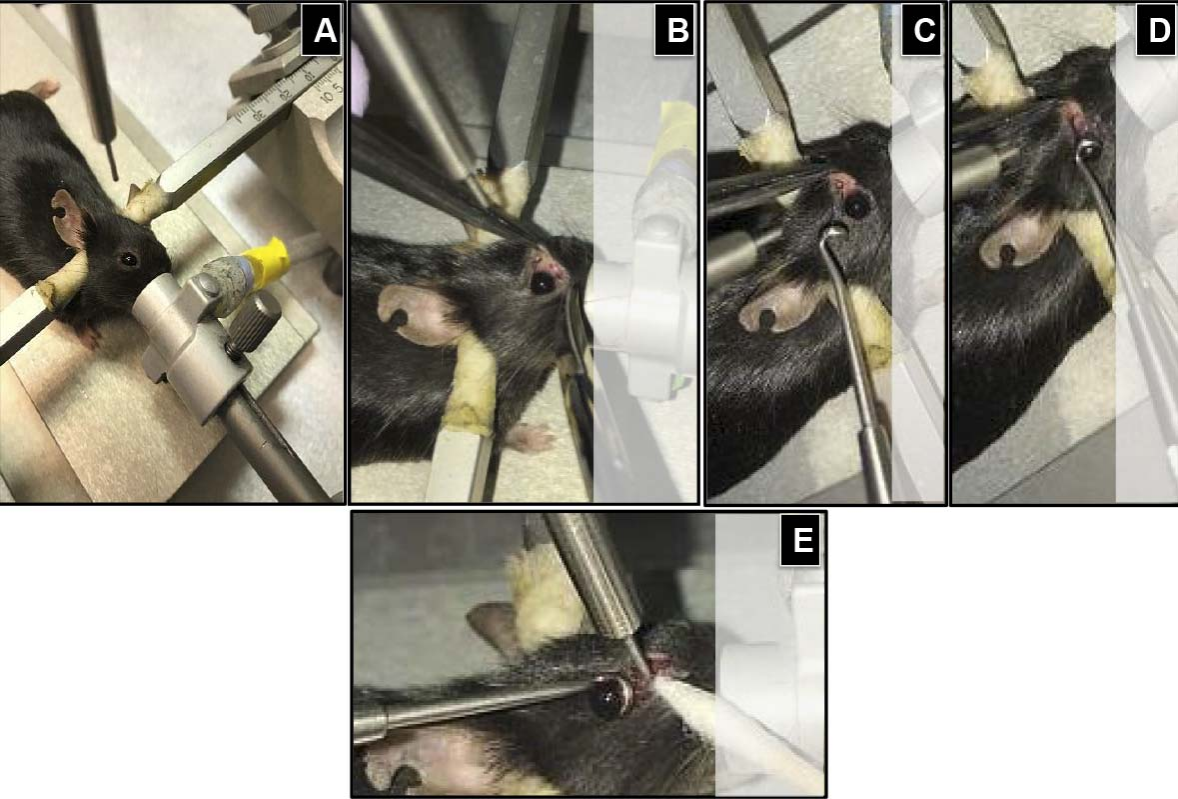
The procedure for controlled ocular impact. (A) The anesthesia was maintained through an inlet tube mounted to a cylindric metallic shield covering the bite plate; (B) by using a pair of blunt laminectomy forceps and scissors, an incision was made at the medial canthus; (C–E) using a noninvasive approach, the eyeball was retracted from the orbital margin; leaving extraocular tissues fully exposed to the controlled impaction from a blunt impactor tip 1 mm in diameter.

**Figure 3:**
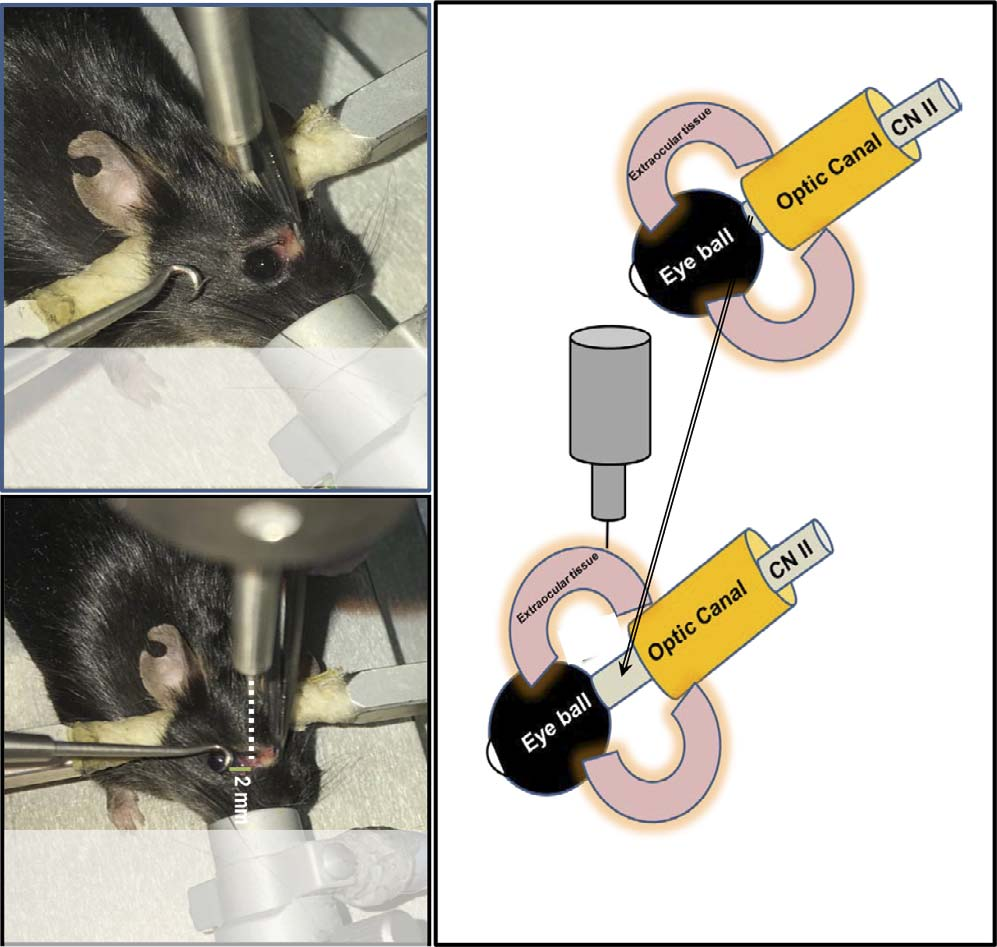
Schematic presentation of the injury site at a distance of 2 to 3 mm from the posterior pole of the globe. The retraction of the eyeball from the orbital margin makes the intraorbital portion of the optic nerve easily accessible and reproducibly injured by the application of controlled impact to the extraocular tissue in the orbital area posterior to the globe.

Each mouse was surgically prepared for the procedure as follows: the mouse was initially placed in an anesthesia induction chamber containing 2% isoflurane vol/vol in 25% oxygen/air mixture, and then transferred to stereotactic frame, where the head was fixed in place using the ear bars and bite plate. The anesthesia was maintained under 2% isoflurane through an inlet tube mounted on a cylindrical metallic shield covering the bite plate (Fig. 2A). By using a pair of blunt laminectomy forceps and scissors, an incision was done at the medial canthus of each anesthetized mouse (Fig. 2B). Special care was taken to avoid bleeding, and if there was any bleeding, a cotton-tipped applicator was gently applied until the bleeding stopped. Then by using a noninvasive retractor (Fig. 2C), the eyeball was retracted from the orbital margin (Fig. 2D), leaving extraocular tissues fully exposed to the controlled impact from a blunt impactor tip with a 1-mm diameter (Fig. 2E). This metallic tip is attached to the PinPoint 3000 instrument and aligned to the injury site at a distance of 2 to 3 mm from the posterior pole of the globe (Fig. 3) to avoid the contact with supraorbital ridge bone. The zero point was established by moving the impactor down until the tip touched the surface of the impact site, then the impact switch was hit to generate the trauma (Supplementary Video S1). The controlled optic trauma was performed at either 2.0- or 3.0-m/sec velocity, with the contusion depth and contusion time remaining constant at 0.6 mm and 100 ms, respectively. The severity of trauma was based on the assessment of the pSTR of the ERG after 2 days of injury. The trauma was performed on the right eye, whereas contralateral left eye remained unaffected and was used as internal control. Another control group included mice that were not impacted nor undergone any surgical intervention and is referred to as naïve control. In our experience, the impactor velocity relative to mouse body weight is the most critical factor for successfully reproducing consistent TON results. At 2 days after the injury, there were statistically significant declines in pSTR amplitudes of injured eyes when compared with their corresponding contralateral controls or those of naïve mice. The impactor velocity was ideally determined for a 20- to 25-g mouse to be between 2 (mild TON) and 3 m/s (moderately severe TON). Impactor velocity of more than 3 m/s may induce maximal retinal function impairment and permanent loss of pSTR. Impactor velocity of less than 2 m/s may cause concussion-like symptoms with no significant difference in ERG.

### ERG Recording

The procedure was as previously described.^23^ Briefly, overnight dark-adapted mice were anesthetized by intraperitoneal injection of a xylazine and ketamine cocktail at doses of 10 and 80 mg/kg, respectively. Temperature was maintained at 37°C by a rectal probe connected to a heating pad. To record ERG responses, pupils were dilated (using Tropicamide [0.5%] and phenylephrine HCl [2.5%]) then silver-coated nylon thread electrodes were carefully positioned on the corneal surface, and a drop of hypromellose was applied to enhance conductivity. For reference electrodes, stainless-steel needles were inserted subcutaneously in each cheek, and a third needle was placed in the tail to serve as a ground electrode. Electrode signals were conducted to a PsychLab EEG8 amplifier (Contact Precision Instruments, Cambridge, MA, USA), whereby raw signals were amplified 10000×, filtered between 0.3 and 400 Hz, and outputs were digitized by a NI-6229 device (National Instruments, Austin, TX, USA). For retinal illumination, 5-ms flashes of varying intensities, generated by a light emitting diode (LED; Lightspeed Technologies, Campbell, CA, USA), were focused through three optic fibers with a 1-mm diameter. Two of these optic fibers were directed to the two eyes, and the third to a photodiode for real-time monitoring of the stimulus timing and intensity. To monitor inner retina functionality, the (“dim”) setup was used, where a neutral density filter with an optical density of 4.0 was placed after the focusing lens, which was positioned to defocus the light onto the optic fibers in order to reduce the transmitted light intensity. With this setup, dim stimuli (down to 3.2 × 10^−8^ lumens [lm] and up to 4 × 10^−6^ lm) were generated. The LED was controlled by Igor Pro 6 software (WaveMetrics, Lake Oswego, OR, USA) with signals sent to the NI-6229 device as voltages. These voltages were recorded redundantly to determine the amplitude and timing of the stimuli (depending on the voltage-to-lm calibrations). The stimulus intensities (3.2 × 10^−8^, 5 × 10^−8^, 9 × 10^−8^, 3 × 10^−7^, 6 × 10^−7^, 1.2 × 10^−6^, and 4 × 10^−6^ lm) were chosen pseudorandomly from a probability density function peaking at the luminances just above threshold, to enhance signal-to-noise ratios in assessing scotopic threshold responses (STRs) as well as to maintain dark adaptation.^23,24^ pSTRs) were measured as the voltage at 110 ms after the flash relative to a baseline taken as the mean over the 500 ms before the flash. Negative STRs (nSTRs) were measured as the negative of the voltage at 200 ms after the flash. Both pSTRs and nSTRs were rectified at 0 μV.

### Western Blot Analysis

Retinas were harvested for Western blot analysis according to a previously described procedure.^25^ Antibodies for Brn3 (1:1000; Abcam, Cambridge, MA, USA), GFAP (1:1000; Cell Signaling Technology, Beverly, MA, USA), Iba-1 (1:1000; Wako Chemicals, Richmond, VA, USA), and GAPDH (1:2000; Cell Signaling Technology) were detected with a horseradish peroxidase-conjugated antibody and enhanced chemiluminescence detection system (Thermo Fisher Scientific, Rockford, IL, USA). Intensity of immunoreactivity was measured by densitometry using ImageJ software (http://imagej.nih.gov/ij/; provided in the public domain by the National Institutes of Health, Bethesda, MD, USA).

### Statistical Analysis

Amplitudes were measured from averaged responses obtained across repeated stimulus trials. Two-factor ANOVA was then used with post hoc *t*-tests corrected for multiple comparisons across intensities by the Holm–Bonferroni method. Data are represented as mean and SEM. For Western blot analysis, two-tailed Student's *t*-test and interquartile range test for outlier exclusion, as described in the figure legends, were used.

## Results

In this model of controlled orbital impact trauma, TON was produced and ranged in severity from mild to severe, depending on the velocity of the impactor. The impact velocity used was either 2.0 or 3.0 m/sec, with the contusion depth and contusion time remaining constant at 0.6 mm and 100 ms, respectively. At these settings, no eye injuries were observed and no mice died as a direct result of contusion.

**Figure 4:**
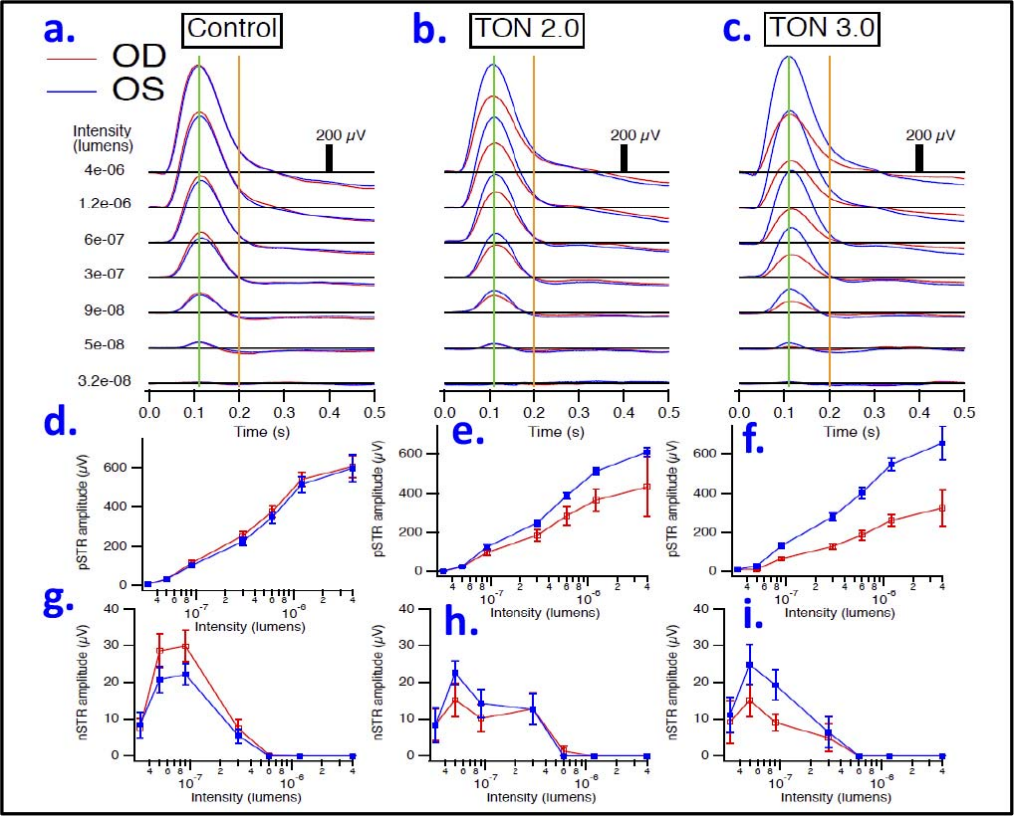
Representative scotopic threshold responses. Averaged responses are shown for control naïve mice (a), traumatic group injured with impactor velocity 2.0 m/s (b), and traumatic group injured with impactor velocity 3.0 m/s (c). Intensities are listed on the left, in lumens. Blue traces are responses obtained from the left eyes, whereas red traces are responses obtained from the right eyes. At 110 ms after the stimulus onset (green vertical lines), pSTRs were measured for each intensity relative to baseline (horizontal black lines) and plotted in (d–f) for each aforementioned group. At 200 ms after the stimulus onset (orange vertical lines) nSTRs were measured at eachintensity, and plotted in (g–i) for each aforementioned group. n = 3–4/group; OD, right eye; OS, left eye.

The postimpact ERG response of the inner retina, specifically the ganglion cells (RGC), has been used to noninvasively assess the visual function impairment and thus the severity of injury following the optic nerve crush as previously published.^23,26^ In mice, the STR, which has pSTR and nSTR components, has been shown to reflect inner retina function and to be more sensitive to the effects of background light than b- and a-waves. Therefore, 2 to 3 days after injury, the mice were dark-adapted overnight and then tested with 5-ms dim flash stimuli of increasing intensities (3.2 × 10^−8^, 5 × 10^−8^, 9 × 10^−8^, 3 × 10^−7^, 6 × 10^−7^, 1.2 × 10^−6^, and 4 × 10^−6^ lm) to detect STRs. As shown in Figures 4a to 4c, the stimulus flash at 0 seconds evoked STRs at the different stimulus intensities. Each experimental group (0.0 [naïve control], 2.0, and 3.0 m/s, respectively) is represented as an average over all animals in that group. Blue traces are responses obtained from the left eyes, whereas red traces are responses obtained from the right eyes. At 110 ms after the stimulus onset (green vertical lines), pSTRs were measured and their amplitudes were quantified for each intensity from the peak to prestimulus baseline (horizontal black lines), whereas at 200 ms after the stimulus onset (orange vertical lines) nSTRs were observed and their amplitudes were quantified as previously described.^23,24^ At lower intensities, the pSTR originates primarily from ganglion cell activity; at higher intensities, the pSTR is occluded by the b-wave, reflecting ON-bipolar cell activity.^24^

Next, the averaged values of the STR (both positive and negative) amplitudes at the seven measured flash intensities were compared between left and right eyes within the same experimental group. Although no significant differences in pSTR amplitudes, at all measured intensities, were observed between left and right eyes in the naïve control group (without trauma, Fig. 4d), these differences were apparent and intensity-dependent within traumatic groups and were greater with the 3-m/sec impact compared with the 2-m/sec impact. At the lowest flash intensity (3.2 × 10^−8^ lm), no statistical differences in the amplitudes of pSTR between left and right eyes were found in any of the TON groups. However, at higher flash intensities these differences became increasingly evident in both TON groups (having lower and higher impactor velocities of 2 and 3 m/s, respectively) but were statistically significant in the latter only. Often dissociable from the pSTR,^23,27,28^ the nSTR, which reflects amacrine cell function in the inner retina,^29^ was affected differently in TON. The amplitude differences of the nSTR between the left and right eyes were not significant among all studied groups at any flash intensity (Figs. 4g–i).

**Figure 5:**
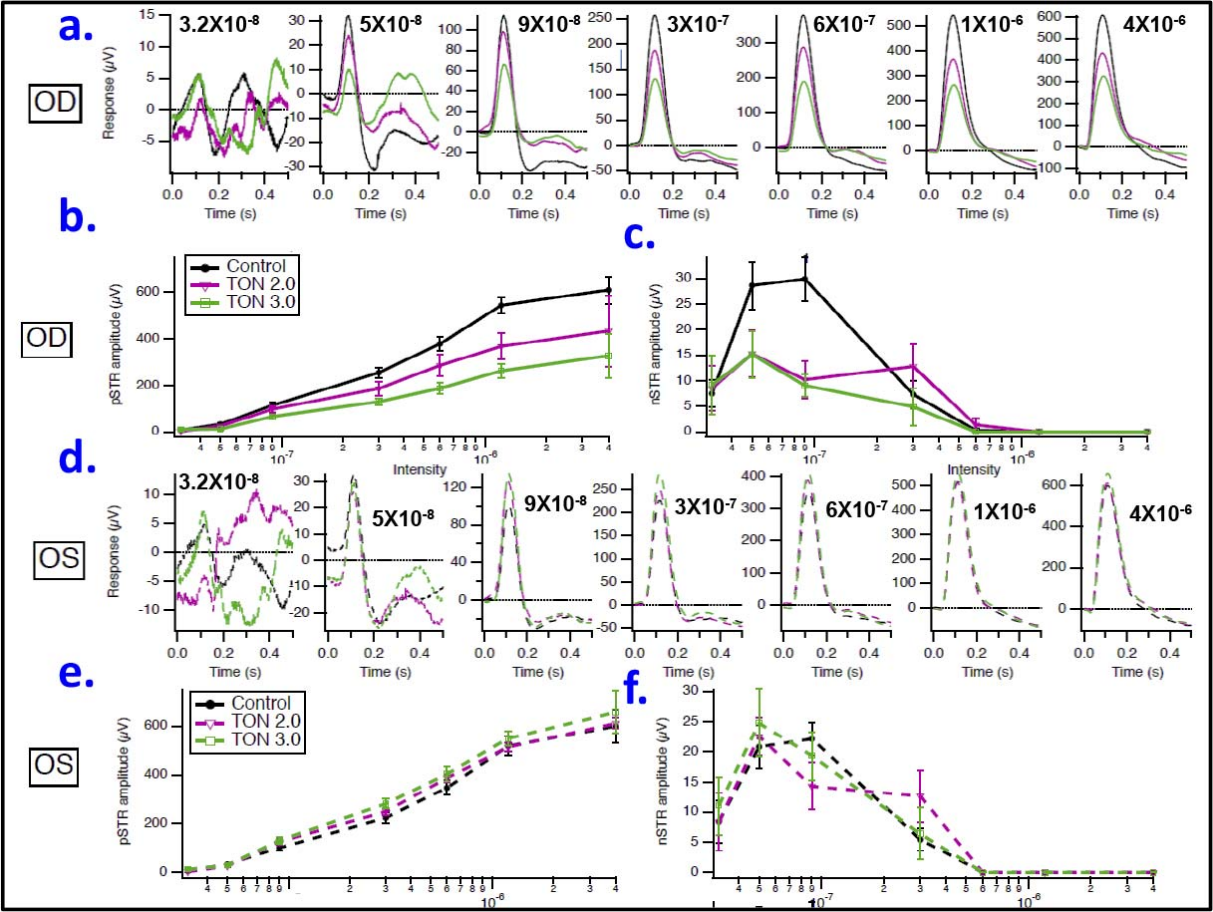
Graded COI causes dose-dependent severities of TON. (a) Averaged pSTRs of traumatized eyes (right eyes), at seven measured intensities, in the two groups of TON with velocities of 2.0 m/s (pink traces) and 3.0 m/s (green traces) superimposed over the corresponding responses obtained from right eyes in the naïve control group (black traces). (b) Dose-dependent decreases in amplitudes of pSTR with increased impactor's velocity. (c) Analysis of nSTR amplitudes did not show a dose-dependent relationship with the impactor velocity despite these amplitudes appearing to be significantly reduced in TON groups over the flash range of (5 × 10^−8^ to 9 × 10^−8^) in comparison with the naïve control group, n = 3–4/group. (d–f) Averaged STRs of contralateral eyes (left eyes), at seven measured intensities, from the two groups of TON with velocities of 2.0 m/s (dashed pink traces) and 3.0 m/s (dashed green traces) were superimposed over the corresponding responses obtained from left eyes in the naïve control group (dashed black traces). Analysis of pSTRs (e) as well as nSTRs (f) did not show a statistically significant effect of the impactor on inner retinal functionality of contralateral eyes in TON studied groups. n = 3–4/group.

Next, in Figure 5, the averaged STRs of the traumatized eyes, at the seven measured intensities, in the two groups of TON with velocities of 2.0 (pink traces) and 3.0 m/s (green traces) were superimposed over the corresponding responses obtained from right eyes in the naïve control group (black traces) to characterize the dose-dependent response to the impactor velocity. Figure 5a shows examples of these individual averaged waveforms, where pSTR peaks are consistently lower in TON groups than in naïve control group. This is evident at all but the lowest intensity. This decrease in pSTR is clearly dependent on the velocity of the impactor, in a dose-dependent manner, as analyzed in Figure 5b. In this figure, the pSTR amplitudes were reduced by approximately 30% and 50% in TON2.0 and TON3.0 groups, respectively, compared with the corresponding pSTRs in the naïve control group as follows: at 9 × 10^−8^ lm (114 ± 14 μV in control, 98 ± 13 μV in TON2.0, and 66 ± 10 μV in TON3.0), 3 × 10^−7^ lm (254 ± 21 μV in control, 186 ± 29 μV in TON2.0, and 129 ± 15 μV in TON3.0), 6 × 10^−7^ lm (377 ± 29 μV in control, 285 ± 48 μV in TON2.0, and 186 ± 24 μV in TON3.0), 1.2 × 10^−6^ lm (543 ± 36 μV in control, 366 ± 56 μV in TON2.0, and 262 ± 29 μV in TON3.0), and 4 × 10^−6^ lm (608 ± 58 μV in control, 433 ± 151 μV in TON2.0, and 326 ± 93 μV in TON3.0). On the other hand, the analysis of nSTR amplitudes did not show a dose-dependent relationship with the impactor velocity, although these amplitudes appeared to be significantly reduced in TON groups over the flash range of (5 × 10^−8^ to 9 × 10^−8^) in comparison with the naïve control group (Fig. 5c).

Next, we sought to further determine whether this model of controlled orbital trauma had an impact on the contralateral eyes. To achieve this, the STRs from the internal control (contralateral left eyes), at the seven measured intensities, from the two groups of TON with velocities of 2.0 (dashed pink traces) and 3.0 m/s (dashed green traces), were averaged and superimposed over the corresponding responses obtained from left eyes in the naïve control group (dashed black traces). Figure 5d shows examples of these individual averaged waveforms, where no difference in pSTR peaks between TON internal controls and naïve control group was seen. Furthermore, the analysis of pSTRs (Fig. 5e) as well as nSTRs (Fig. 5f) did not show a statistically significant effect of the impactor on inner retinal functionality of contralateral eyes in TON studied groups.

**Figure 6:**
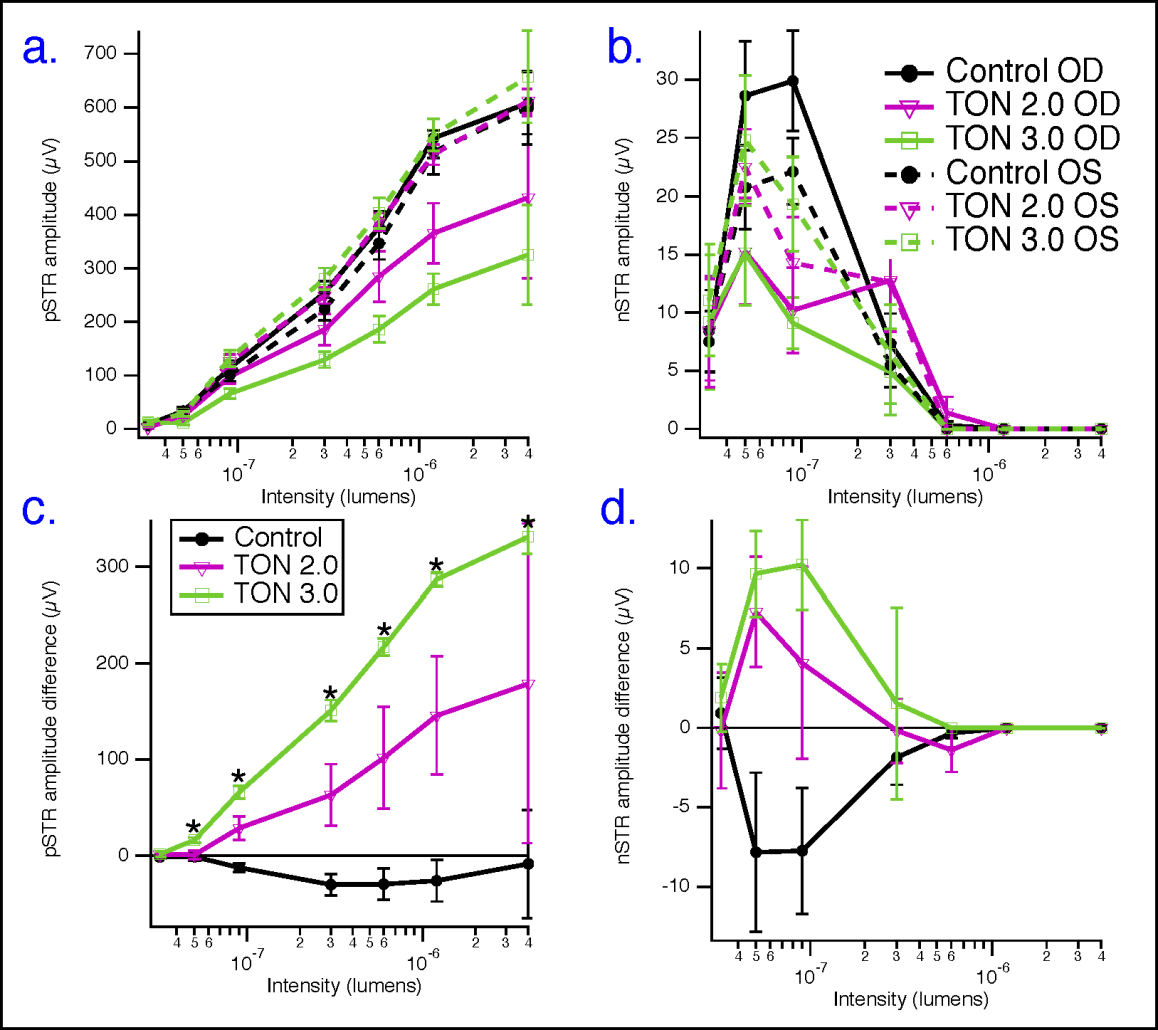
Normalization of STR amplitudes. (a) Pooled data, including both eyes from all animals, for pSTR analysis; (b) pooled data for nSTR analysis. Responses from right and left eyes of naïve control mice are represented as solid- and dashed-black, respectively; responses from right (traumatized) and left (contralateral control) eyes of TON2.0 mice are represented as solid- and dashed-pink, respectively; responses from right (traumatized) and left (contralateral control) eyes of TON3.0 mice are represented as solid- and dashed-green, respectively. (c, d) Data normalized between animals across different runs by subtracting the amplitude of pSTRs or nSTRs obtained from each traumatized eye from its corresponding amplitude obtained from the contralateral eye (internal control) in the same animal and represented as averaged ΔpSTRs or ΔnSTRs, respectively, at each flash intensities; green represents TON3.0 group, whereas pink represents TON2.0 group. n = 3–4/group; OD, right eye; OS, left eye. *Difference at the P < 0.05 level after correcting for multiple comparisons.

Because of animal to animal variability, interanimal and intra-animal data were pooled in Figures 6a and 6b for subsequent pSTR and nSTR analyses, respectively. Amplitudes of pSTRs or nSTRs were normalized between animals across different runs by subtracting the amplitude of pSTRs or nSTRs obtained from each traumatized eye from its corresponding amplitude obtained from the contralateral eye (internal control) in the same animal and represented as averaged ΔpSTRs or ΔnSTRs at each flash intensity (Figs. 6c and 6d, respectively). As shown in Figure 6c, ΔpSTRs in TON2.0 (pink) and TON3.0 (green) first underwent a small increase at lower flash intensities, then a sharp increase at middle- and high-flash intensities. Additionally, ΔpSTRs showed a strong and dose-dependent association with the applied impactor velocity, whereas ΔnSTRs did not show that pattern of dose-dependent relationship with the impactor velocity (Fig. 6d). Taken together, ΔpSTRs rather than ΔnSTRs could be used as a potential indicator representing the extent of ganglion cell dysfunction in the optic nerve trauma.

**Figure 7:**
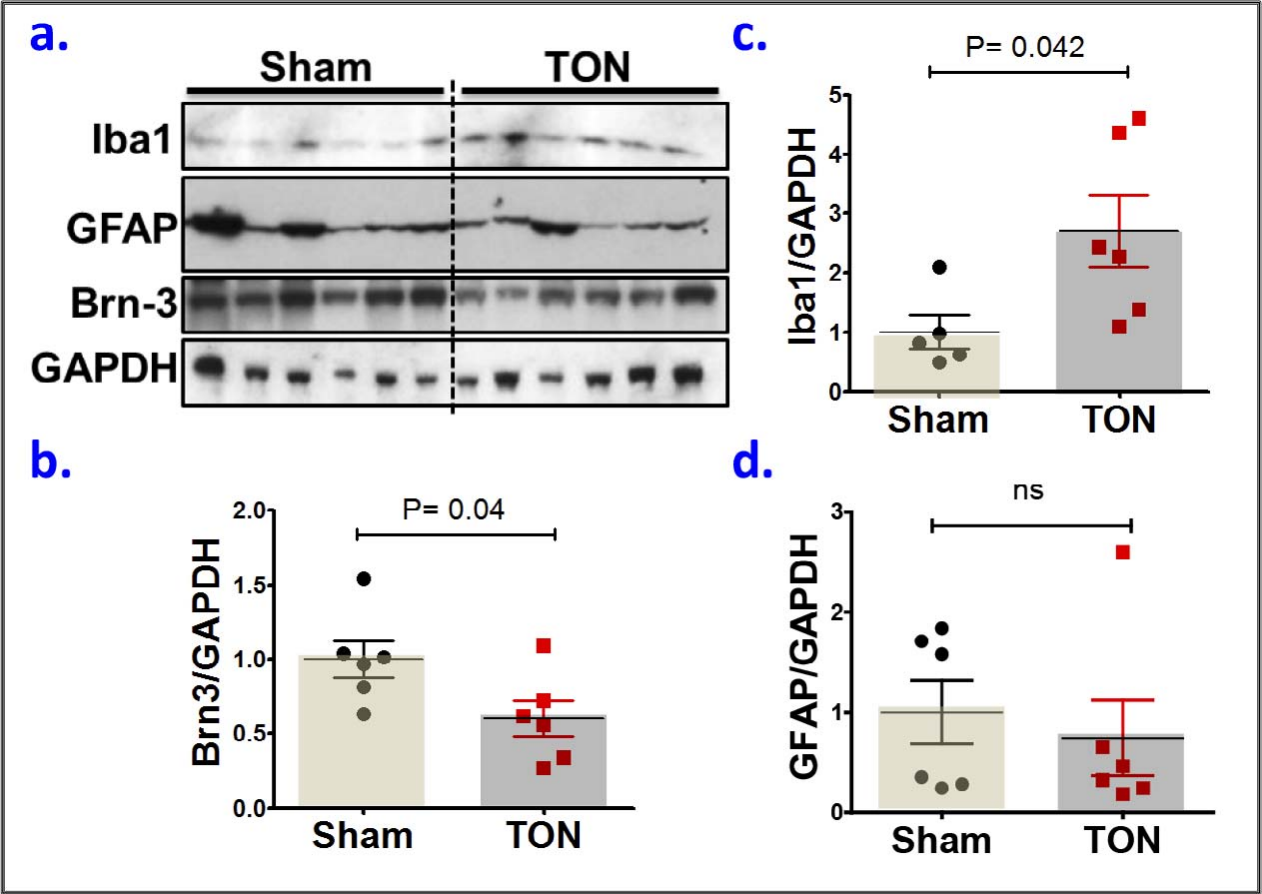
COI induces RGC loss and microglial activation. (a) Western blot for Brn3, ionized calcium-binding adapter molecule 1 (Iba-1), and GFAP, markers for RGC, microglia, and Müller glial cells, respectively. (b–d) Densitometric analysis of Brn3, Iba-1, and GFAP, respectively. Each symbol (b–d) represents an individual mouse; small horizontal lines indicate the mean ± SEM. ns, nonsignificant. P > 0.05, unpaired, two-tailed Student's t-test, interquartile range test for outlier exclusion.

In further characterization of this model on the cellular level, we examined the response of neurons, glia, and microglia to the impact. As shown in Figures 7a and 7b, there was a significant loss of Brn-3, a ganglion cell marker, in the TON group compared with TON internal controls. This loss of Brn-3 was associated with the activation of microglia, but not Müller glia, in response to the ocular trauma. These are illustrated by the significantly increased expression of Iba1, a microglia marker, in TON group compared with TON internal controls (Figs. 7a, 7c), whereas the expression of GFAP, a Müller glia marker, was similar in both groups (Figs. 7a, 7d).

## Discussion

The controlled impact model was initially developed to study traumatic brain injury, which is now referred to as controlled cortical impact (CCI). The precise controlling and reproducibility of CCI led to its popularity among the commonly used models of traumatic brain injury.^30^ In this study, we modified and optimized CCI to adapt it for the use in studying TON, wherein the optic nerve is reproducibly exposed to a graded controlled impact of known velocity. The key features of this new modified model “controlled orbital impact (COI)” are (1) the injury parameters (velocity, contusion depth, and contusion time) are quantifiable and can all be managed to generate a wide range of TON severities; (2) a reproducible electrophysiology endpoint of diminished ERG response can be achieved in a clinically relevant mouse model of closed ocular injury without the interference of surgical variability and tissue damage associated with optic nerve crush models; (3) the contralateral eye showed no significant difference to the eyes of naïve mice, allowing its use as an internal control to minimize interindividual variability among mice; and (4) the occurrence of injury-associated mortality and/or ocular co-morbidity, including cataract and corneal edema, is rare as compared with other models of TON, such as ocular blast injury model.^20^

The pathogenesis of TON is uncertain, with many possible mechanisms accountable for visual loss.^31^ The orbital and canalicular portions of the optic nerve are the sites most frequently involved in indirect optic nerve injuries from blunt trauma to the frontal or midfacial areas.^32,33^ Therefore, an effective experimental model of indirect TON should transmit the force either to intraobital or intracanalicular portions of the optic nerve in a quantifiable, reproducible, and adjustable manner. However, the intracanalicular portion is susceptible to compression or direct transection with the fixed sharp edge of the falciform dural fold at the edge of the optic canal from small lesions.^20^ This susceptibility may cause unpredictable permanent nerve damage and significant variations in outcome measurements in terms of graded dose-response relationship after trauma. Furthermore, the possibility of transmission of the trauma to the intracanalicular portion of the optic nerve of the contralateral eye is more likely due to the anatomic closeness of optic nerves in the mouse, which precludes the use of contralateral eye as an internal control in some TON models, such as SI-TON.^21^ On the other hand, the intraorbital portion of the optic nerve is accessible and can be reproducibly injured, but to date there is no standardized model to deliver such injury in a quantifiable and graded manner. On that basis, we have developed and characterized COI as a new mouse model for TON, whereby the application of controlled impact to the extraocular tissue in the orbital area posterior to the globe produces a graded deficit in RGC electrophysiological functions. Taking advantage of the optic nerve being composed of RGC axons, its injury induces RGC loss and functional deficits.^34,35^ We used pSTR as a monitoring tool for optic nerve injury because pSTR is generated in large part by RGC and is able to detect the early RGC dysfunction.^16^

Another key feature of the COI model is that the impact is directed to soft tissues at the extraocular site, which does not include the supraorbital ridge, so that the possibility of bone fracture as well as transmission of the effect of trauma to the contralateral eye is minimal. This has been supported by our results that showed no significant difference between ERG responses of the contralateral eyes of the impacted mice and that of naïve mice. As such, the standardized procedure in our model can be normalized to overcome the interindividual variability associated with experimental conditions by measuring (ΔpSTR) between the traumatized eye and its corresponding contralateral eye in the same mouse.

In further characterization of the COI model, we examined the expression of RGC marker (Brn3) and reactivity of glia cells, which normally respond to tissue injury caused by physical trauma, chemicals, or infection.^36^ Our results have shown a significant decrease in Brn3a expression (measured by Western blot) as early as 3 to 5 days following TON, in agreement with previous Brn3 analyses in optic nerve–injured retinas.^13,37^ Furthermore and consistent with previous TON models,^21,38,39^ a similar microglia activation, represented by the increased Iba-1 expression, has been observed in our model. Activated microglia cells have cytotoxic and phagocytic capabilities to destroy foreign materials and to engulf dead cells. However, prolonged activation of microglial cells has been reported to be associated with inflammation and thereby neuronal cell loss seen in TON.^39^

In summary, we have developed a mouse model of COI with characteristics similar to those seen in patients with traumatic ocular injury, including partial loss of RGC function and microglia activation. This model is minimally invasive with advantages of simplicity, precision control, reproducibility, and ability to cause different severities of TON. This model delivers a controlled impact in the orbital area posterior to the globe, which produces a graded quantifiable deficit in RGC functions with no accompanying ocular morbidity, mortality, or fracture. Thus, this model overcomes some limitations of prior TON mouse models and provides an innovative platform to identify therapeutic targets for neuroprotection and/or neurorestoration following traumatic ocular injury.

## Supplementary Material

Supplement 1
